# A modified preauricular and transmandibular approach for surgical management of osteosarcoma of the mandibular condyle within the masticator space and infratemporal fossa: a case report

**DOI:** 10.1186/s13256-019-1975-1

**Published:** 2019-03-12

**Authors:** Tadahide Noguchi, Yasushi Sugiura, Naruo Okada, Yoshiyuki Tsuchiya, Jun-ichi Hyasaka, Ken-ichi Sasaguri, Shunji Sarukawa, Akifumi Fujita, Yusuke Amano, Yoshiyuki Mori

**Affiliations:** 1 Department of Dentistry, Oral and Maxillofacial Surgery, Jichi Medical University, 3311-1 Yakushiji, Shimotsuke-shi, Tochigi, 329-0498 Japan; 2 Department of Plastic Surgery, Saitama Medical University, International Medical Center, Yamane 1397-1, Hidaka-city, Saitama, 350-1298 Japan; 3 Department of Radiology, Jichi Medical University, 3311-1 Yakushiji, Shimotsuke-shi, Tochigi, 329-0498 Japan; 4 Department of Pathology, Jichi Medical University, 3311-1 Yakushiji, Shimotsuke-shi, Tochigi, 329-0498 Japan

**Keywords:** Osteosarcoma, Mandibular condyle, Masticator space, Preauricular approach, Transmandibular approach

## Abstract

**Background:**

Osteosarcomas of the head and neck region are rare entities that comprise < 10% of all osteosarcomas. Multimodality treatment of patients with osteosarcoma is well-established for osteosarcoma in long bones, and the benefits of chemotherapy in long bones are clearly known. However, there is no consensus regarding the effects of chemotherapy in cases of head and neck osteosarcoma. The prognostic factor for head and neck osteosarcoma is complete tumor resection with negative margin, which is a radical surgery. However, a clear margin may be difficult to achieve in the head and neck region.

**Case presentation:**

We present a case of a 69-year-old Japanese woman who developed osteosarcoma of the condyle within the masticator space and infratemporal fossa, which was treated with radical surgery using a modified preauricular and transmandibular approach. Although we recommended adjuvant treatment after surgery, the patient refused this treatment. There was no evidence of local recurrence or distant metastasis through 30 months of follow-up.

**Conclusions:**

Our modified preauricular and transmandibular approach allowed access to the masticator space and infratemporal fossa, thereby increasing complete resection of the tumor and resulting in minimal functional and cosmetic deficits.

## Background

Osteosarcomas at all sites comprise approximately 40–60% of primary malignant bone tumors [1]. Osteosarcomas of the head and neck region are rare entities that comprise < 10% of all osteosarcomas [2–4]. Multimodality treatments of patients with osteosarcoma have been well-established for osteosarcoma in long bones [5]. Chemotherapy for osteosarcoma in long bones is administered as induction. After surgery, the specimen is examined, and histological response is determined, whether or not chemotherapy is administered [5]. Although a few reports have shown that neoadjuvant and adjuvant chemotherapy contributes to improved survival rate in patients with osteosarcoma in the head and neck [6], there is no consensus regarding the effects of chemotherapy in cases of head and neck osteosarcoma. A recent study failed to show a benefit of chemotherapy in head and neck osteosarcoma [7]. The prognostic factor in head and neck osteosarcoma is complete tumor resection with negative margin, which constitutes radical surgery [6–10]. However, a clear margin may be technically difficult to achieve in the head and neck region, particularly in the masticator space and/or infratemporal fossa, because the surgical field of vision is poor, and it is difficult to control bleeding.

In our patient, we performed surgical management via a modified preauricular and transmandibular approach for osteosarcoma of the mandibular condyle that reached the masticator space and infratemporal fossa. This method makes it easy to approach from the glenoid fossa to the infratemporal fossa with a wide field of view by adding a temporal incision and tracing the facial nerve to the conventional preauricular and transmandibular approach.

## Case presentation

A 69-year-old Japanese woman was referred to our department in August 2015, complaining of swelling in the right preauricular region. She had shown a mass lesion of the right lung on a chest x-ray and was referred to the university hospital. Fluorodeoxyglucose-positron emission tomography (FDG-PET) examination showed accumulation indicative of a pulmonary lesion and a temporomandibular joint lesion. The temporomandibular joint lesion had been enlarging but was otherwise asymptomatic. Her medical history included treatment for pulmonary tuberculosis at the age of 13 and surgery to remove breast cancer at the age of 53.

On examination, a tender mass in the right preauricular region was palpable. The chin of the mandible was deviated to the right side during mouth opening (mandibular maximum mouth opening, 41 mm). Facial nerve function and mandibular nerve were intact. There was no indication of cervical lymphadenopathy.

**Fig. 1:**
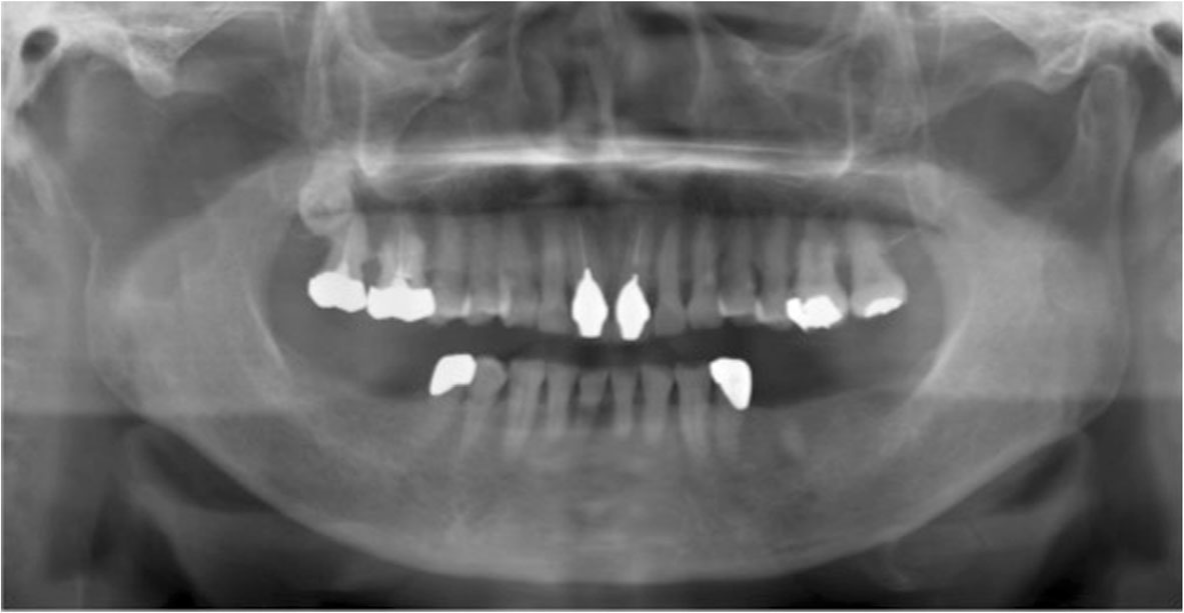
Panoramic radiographic examination showing resorption of the right mandibular condyle to the ramus

**Fig. 2:**
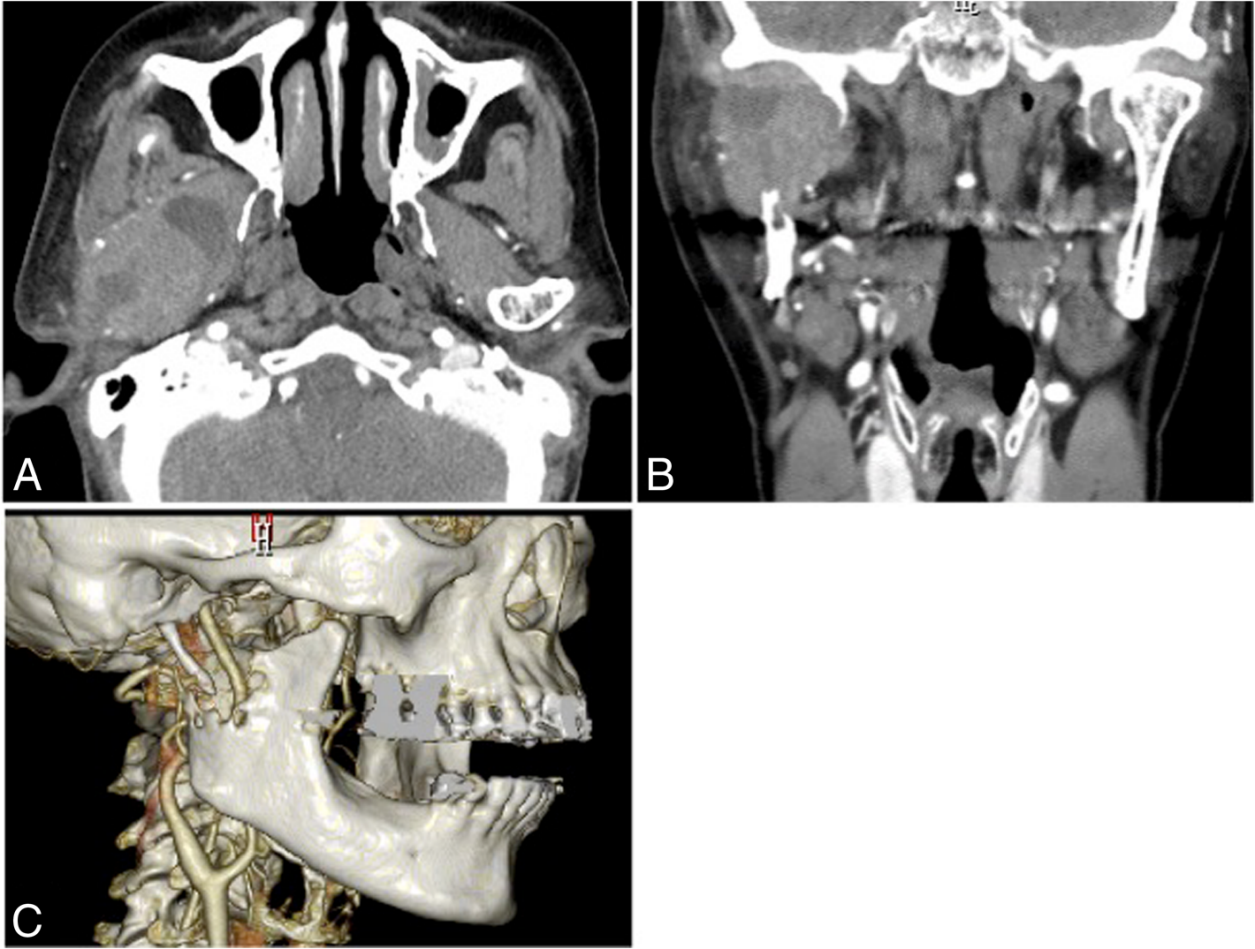
Computed tomography (CT) showing destruction of the right mandibular condyle and a large mass lesion, with margin enhanced in the masticator space. A cystic lesion was present inside the tumor mass. **a** Axial plane. **b** Coronal plane. **c** Three-dimensional CT

**Fig. 3:**
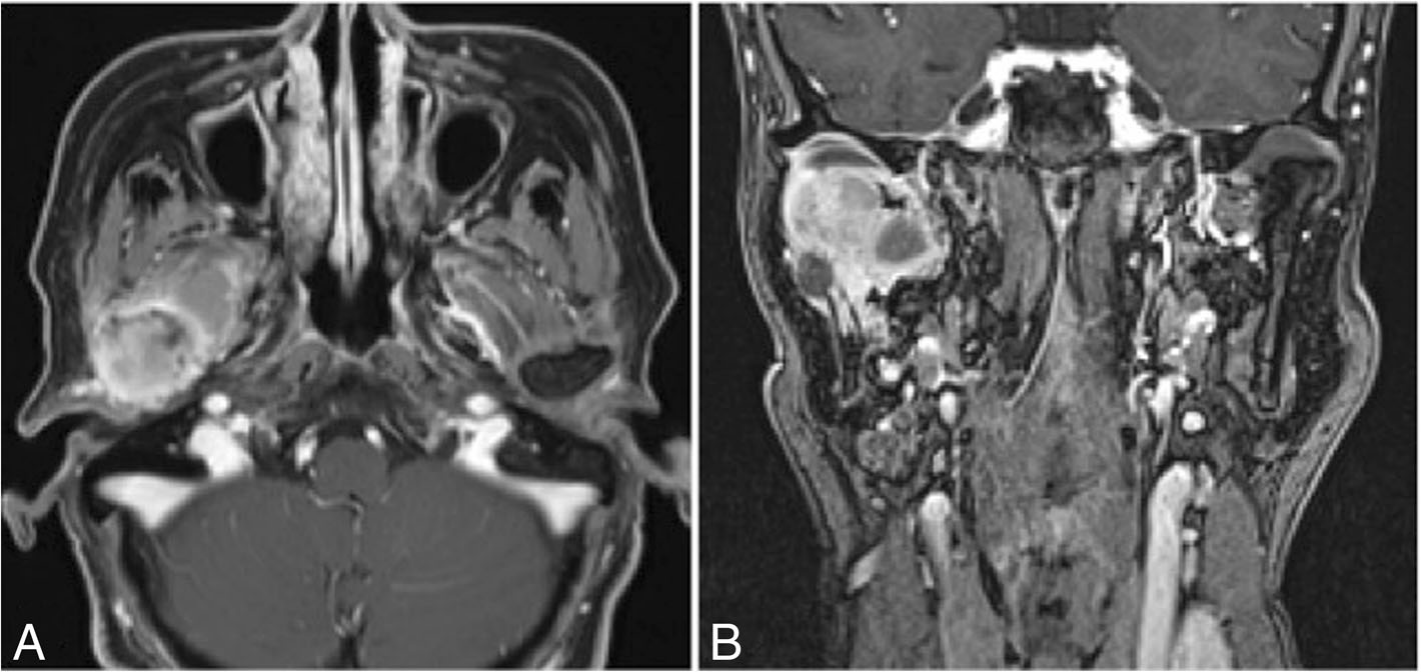
T1-weighted magnetic resonance imaging showing an enhanced mass lesion in the right masticator space. Because some portions of the mass lesion showed high intensity in T2-weighted images, cystic lesions were suspected with changes in blood flow or retention of high-protein liquid  **a**. Axial plane, **b**. Coronal plane

A panoramic radiographic examination showed resorption of the right mandibular condyle to the ramus (Fig. 1). Computed tomography (CT) showed destruction of the right mandibular condyle and a large mass lesion with enhanced margin in the masticator space; a cystic lesion was present inside the tumor mass. Three-dimensional CT was useful to understand the bone resorption findings of the mandibular condyle. There was no finding of metastatic cervical lymph node (Fig. 2). T1-weighted magnetic resonance imaging showed an enhanced mass lesion in the right masticator space. Because some portions of the mass lesion showed high intensity in T2-weighted images, cystic lesions were suspected to exhibit changes of blood flow or retention of high-protein liquid. Tumor development was not observed in the articular disk of the temporomandibular joint (Fig. 3). FDG-PET revealed abnormal FDG uptake in the right submandibular condyle and masticator space.

**Fig. 4:**
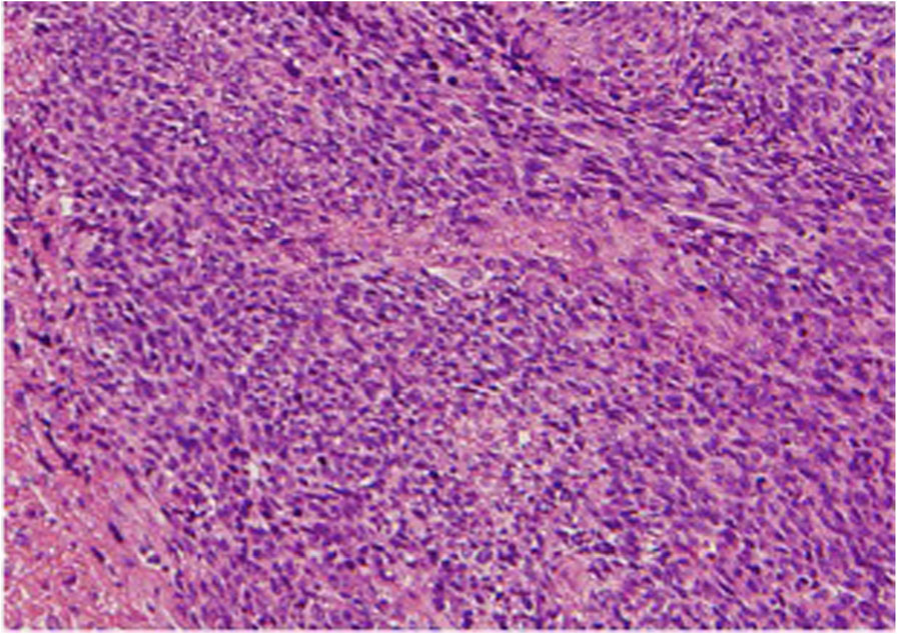
Histopathologically, much of the tumor was composed of proliferative, atypical, spindle-shaped cells. Some tumor cells showed increasing mitotic changes and a high degree of atypia (H&E stain, 100× magnification)

A malignant tumor was suspected after analysis by various modalities. Therefore, we performed an incisional biopsy via preauricular incision. Histopathologically, the tumor was largely composed of proliferative, atypical, spindle-shaped cells. Some tumor cells showed increasing mitotic change and extreme atypia (Fig. 4). The histopathological findings of biopsy suggested spindle-cell sarcoma. Tumor resection was performed with the patient under general anesthesia via a combined preauricular and transmandibular approach to the masticator space and infratemporal fossa.

### Modified preauricular approach

**Fig. 5:**
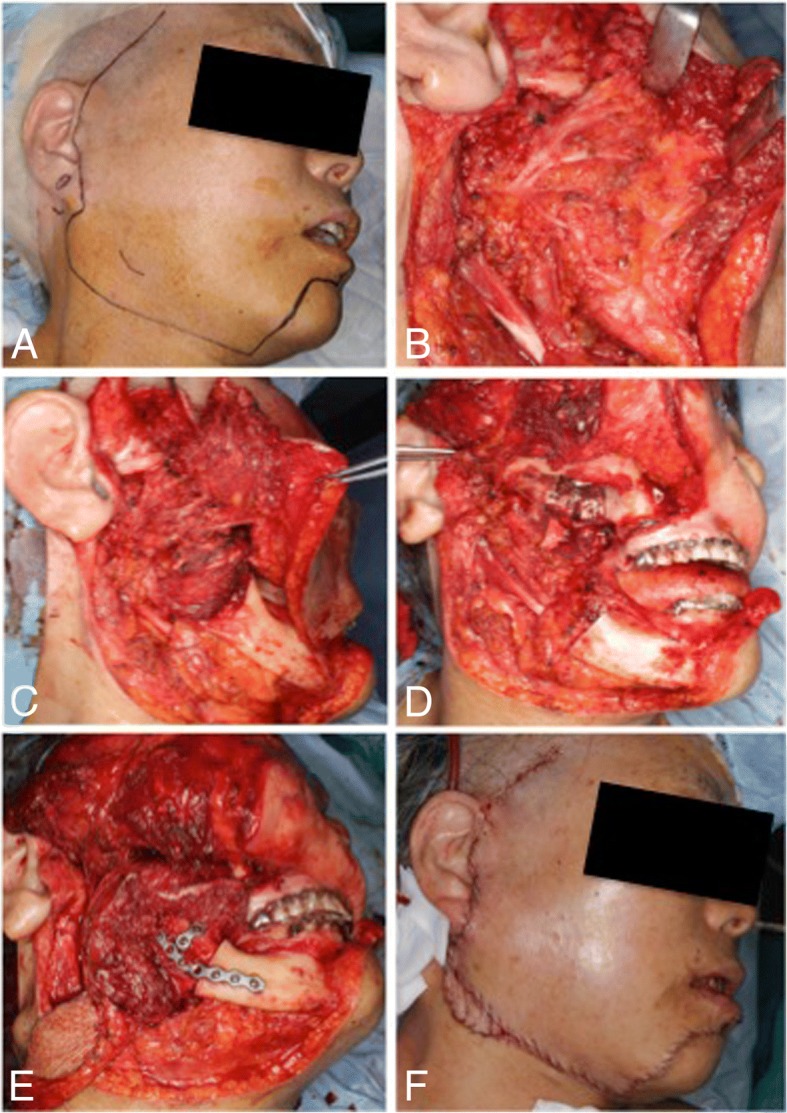
Surgical procedure. **a** Incision line. **b** Facial nerve trace. **c** Facial nerve preservation. **d** After tumor resection. **e** Reconstruction of fibula. **f** After suture

After preauricular temporomandibular incision, the superficial temporal fascia and temporal fascia were elevated. The facial nerve (temporal and zygomatic branches) was protected by the fascia (Fig. 5a).

### Tracing of the facial nerve (Fig. 5b)

The facial nerve trunk was identified, and its branch was traced according to the conventional method.

### Mandibulectomy

A midline lip-splitting incision was connected to the submandibular incision and accessed the anterior mandibular ramus. The lip-split incision traversed to the periosteum of the mandible; the periosteum dissector was used to elevate the soft tissues of the mandible, and the lateral mandible was exposed. The branch of the facial nerve was traced continuously with the skin flap, and the masseter muscle and deep parotid gland were dissected on the tumor side (Fig. 5c). The mandible was resected to within ≥ 20 mm from the primary tumor on the anterior of the mandibular ramus. The masseter muscle was divided at the lower edge of the zygomatic arch; further, the temporal muscle was divided horizontally at the height of the zygomatic arch to reach the side of the temporal bone. Excision was performed on the temporal bone to the base of the pterygoid process, and an osteotomy was performed continuously from the maxilla to the pterygoid process. Osteotomy was performed via ultrasonic scalpel. Then, deep excision was performed on the skull base until the foramen ovale was reached. The mandibular branch (VIII) of the trigeminal nerve was resected at the foramen ovale, and bone wax was used to fill the foramen ovale when hemostasis was achieved. The tumor was excised in bulk with the surrounding tissue (Fig. 5d). Especially, the glenoid fossa was close to the primary tumor site; however, it was excised including the joint disc, and the glenoid and infratemporal fossa were excised, including the periosteum of the skull base. For that reason, the margin was considered complete, and we did not perform the histopathological evaluation intraoperatively.

The surgical defect was reconstructed with a free vascularized fibula with skin paddle (Fig. 5e). The occlusion was performed with intermaxillary wire fixation for 1 week postoperatively. There were no abnormal findings during the postoperative course with complete healing. Right-sided facial nerve dysfunction appeared immediately after surgery.

**Fig. 6:**
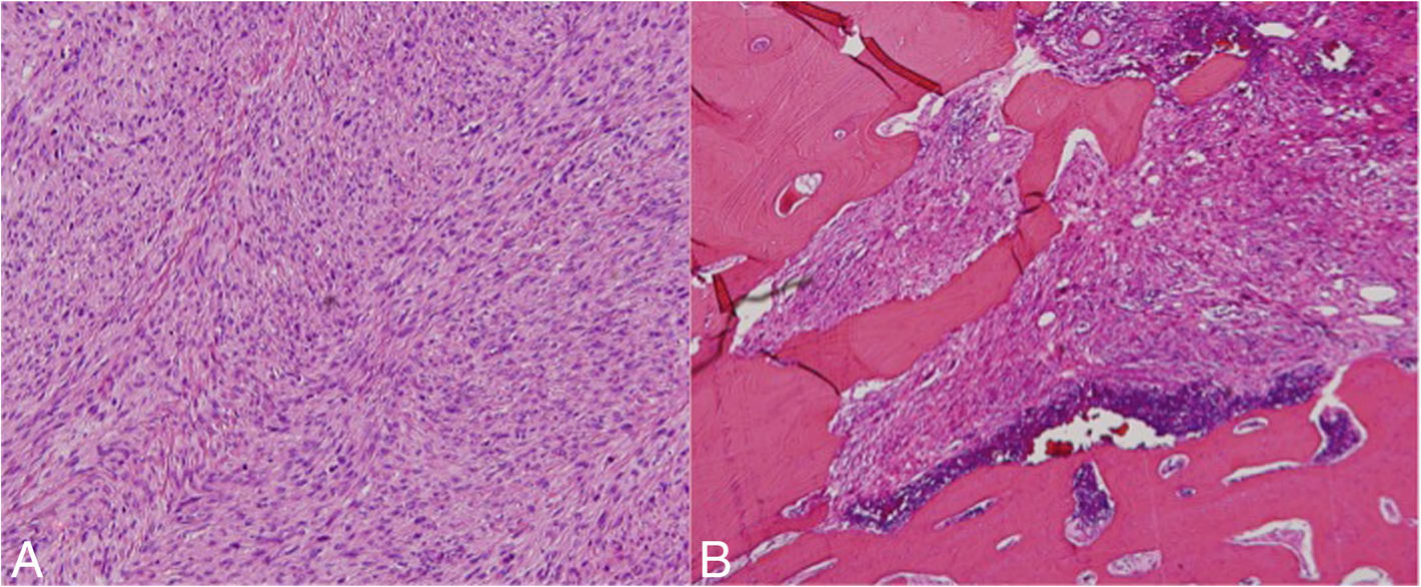
H&E staining. **a** The resected specimen was almost identical to the histological findings of the biopsy. **b** It involved periosteal reaction, corresponding partially to Codman’s triangle. The magnification is 100× in (**a**) and 40× in (**b**)

The resected specimen exhibited nearly identical histological findings as observed in the biopsy. Because it involved a periosteal reaction corresponding partially to Codman’s triangle, the tumor was thought to have derived from bone (Fig. 6). Immunohistochemical analysis showed positive staining for vimentin, MIB-1 index (40–80%), desmin, α-smooth muscle actin, Bcl-2, neuron-specific enolase, and S-100; it showed negative staining for AE1/AE3, caldesmon, and CD34. Thus, the final diagnosis was osteosarcoma (fibroblastic). The resection margin was negative for tumor. After excision of the mandibular tumor, excision of lung cancer was performed under thoracoscopy by a respiratory surgeon in our hospital. Although we recommended adjuvant treatment after surgery, the patient refused this treatment.

**Fig. 7:**
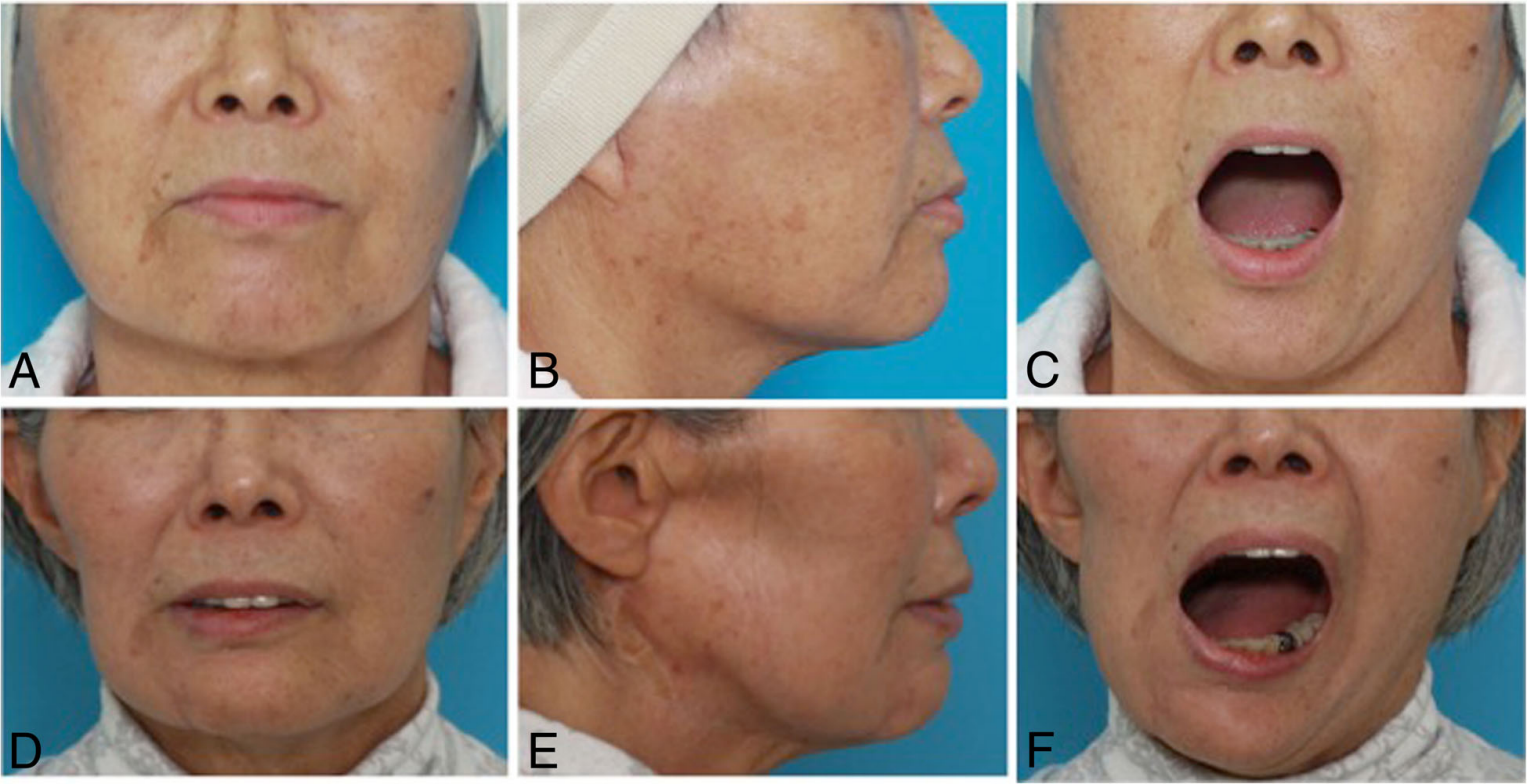
Preoperative (**a**, **b**, **c**) and 27 months postoperative (**d**, **e**, **f**) images

**Fig. 8:**
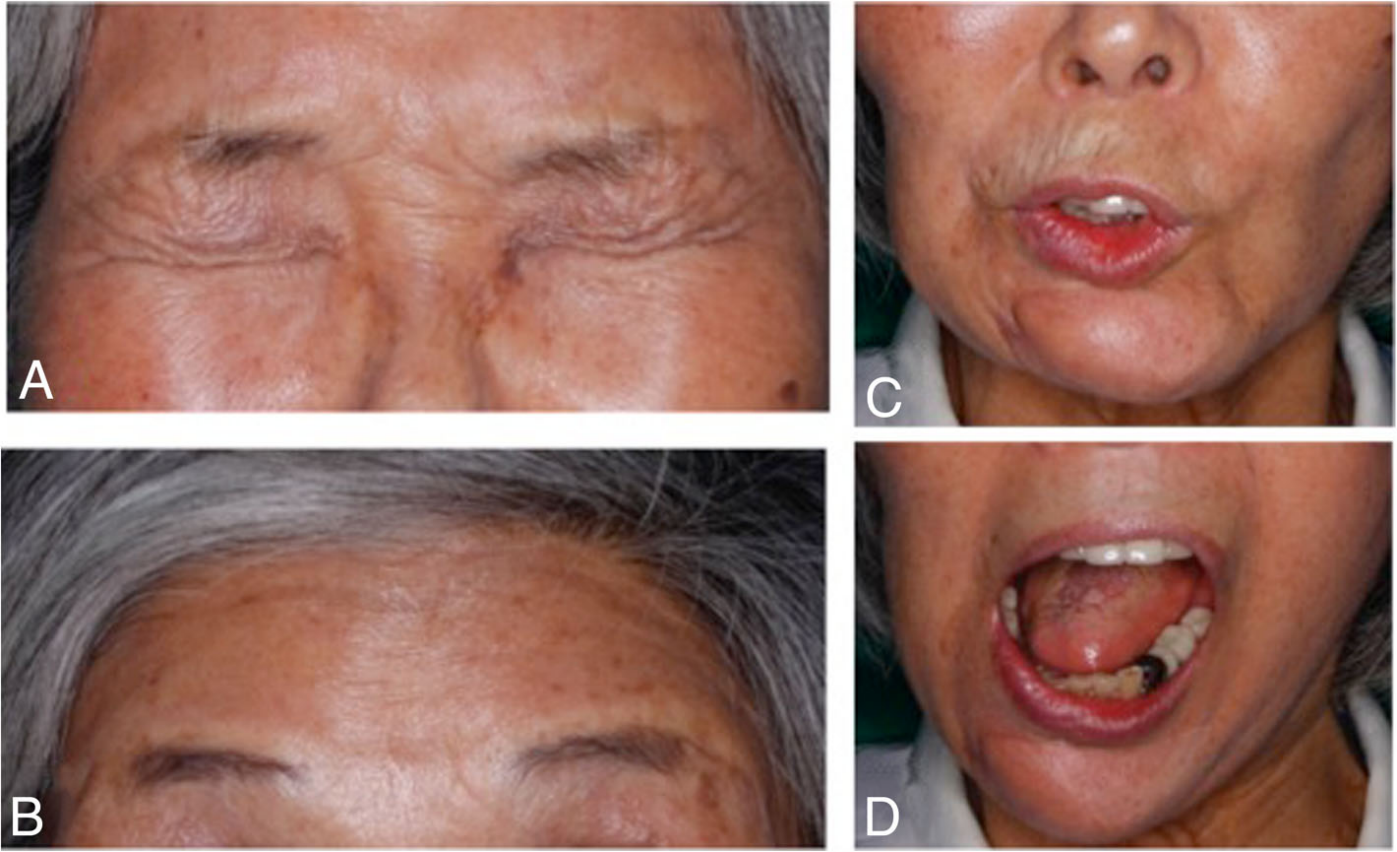
Postoperative images of facial function. **a** Closed eyes. **b** Wrinkling of forehead. **c** Whistling motion. **d** Mouth opening

There has been no evidence of local recurrence or distant metastasis through 30 months of follow-up. The chin of the mandible deviates to the right side during opening (maximum mouth opening, 40 mm). Centric occlusion has not changed. Facial nerve dysfunction gradually improved and became mild according to House-Brackmann Scale evaluation (Figs. 7 and 8).

## Discussion

The most important prognostic factor for osteosarcoma in the head and neck is complete tumor resection with negative margin in radical surgery [6–10]. However, a clear margin may be technically difficult to achieve in the head and neck region, particularly in the masticator space or infratemporal fossa. The masticator space and infratemporal fossa contain the terminal branches of the maxillary artery, pterygoid plexus, and mandibular nerve (V3). Complete tumor resection is difficult with surgery, because the surgical field of vision is poor, and it is difficult to control bleeding in this region. There are anatomical complexities in the head and neck, and various considerations are necessary for surgery, relative to other tumor sites, such as functional and aesthetic impairment.

Regarding surgical margins, there have been reports suggesting the establishment of a margin of 2 cm of bone after effective preoperative chemotherapy [11]; the safe recommended margin in osteosarcoma of the extremities is 3 cm [12]. In head and neck sarcoma, a clear surgical margin of at least 1 cm has been recommended for anatomical reasons [13]. Therefore, 2-cm bony margins and at least 5-mm soft tissue margin have been recommended when reconstructive surgery is possible [12].

In our patient, because the tumor was located in the masticator space and infratemporal fossa and had originated from the mandibular condyle, a modified preauricular and transmandibular approach was selected for complete *en bloc* resection of the tumor and preservation of the facial nerve without cervical branching. There have been various reports regarding approaches to tumors in the infratemporal fossa [14–16]. This surgical approach allows complete exposure of the glenoid fossa and infratemporal fossa, where access is not blocked by the facial nerve. The lateral approach to the infratemporal fossa allows wide exposure of the surgical field, shorter depth of work, and adequate control of bleeding.

Excision using this approach can entirely remove the tumor, including the medial and lateral pterygoid muscle, mandibular ramus, posterior of maxilla, and pterygoid process, with the skull base as the upper border of the surgical margin. Sacrificing the mandibular ramus above the angle of the mandibular condyle allows a wide approach to the glenoid fossa and lower portion of the infratemporal fossa. In addition, the temporal muscle can be dissected from the lateral side and exposed to the infratemporal fossa under direct visualization. Then, the foramen ovale and foramen spinosum can be exposed, and dissection of the mandibular nerve (V3) and treatment of the oval plexus and middle meningeal artery can be performed. It is advantageous for hemostatic treatment to be performed under direct view.

This approach has the following advantages:

1. The facial nerve and its branches (with the exception of the cervical branch) may preserved.
2. Exposure of the glenoid fossa, pterygopalatine fossa, and infratemporal fossa can be readily obtained.
3. The mandibular division of the trigeminal nerve and the pterygoid plates can be exposed.
4. Flaps for reconstruction are available within the surgical field.

This approach can also be applied to the resection of maxillary cancer that has developed backward, such as within the pterygoid muscle and process.

Disadvantages include that the result is not good aesthetically, because there is an incision in the median of the lower lip; however, by extending the incision of the temporal and submandibular regions, this lower lip incision may be avoided.

This method makes it easy to approach from the glenoid fossa to infratemporal fossa with a wide field of view by adding a temporal incision and tracing the facial nerve to the conventional preauricular and transmandibular approach.

This approach is a suitable method for cases requiring complicated excision of temporomandibular joint, masticatory muscles, and pterygoid process in tumors extending from the glenoid fossa to the infratemporal fossa, and it can be suppressed to minimal functional and cosmetic deficits, including preservation of the facial nerve.

## Conclusions

A clear margin may be technically difficult to achieve in the head and neck region, particularly in the masticator space and/or infratemporal fossa, because the surgical field of vision is poor, and it is difficult to control bleeding. This modified preauricular and transmandibular approach allows access to the masticator space and infratemporal fossa, as shown by anatomical dissection, thereby increasing the complete resection of the tumor and resulting in minimal functional and cosmetic deficits.

## Abbreviations

CT: Computed tomography
FGD-PET: Fluorodeoxyglucose-positron emission tomography
